# Expanding the Phenotypic Spectrum of Degcags Syndrome: Novel Craniofacial and Oral Findings

**DOI:** 10.1111/scd.70232

**Published:** 2026-08-08

**Authors:** Jeferson Paiva, Lucas Batista‐Fontes, Janaína Taíza Araújo de Jesus, Kelly Fernanda Molena, Raquel Assed Bezerra da Silva, Kranya Victoria Diaz‐Serrano, Francisco Wanderley Garcia de Paula‐Silva, Alexandra Mussolino de Queiroz, Fabrício Kitazono de Carvalho

**Affiliations:** ^1^ Department of Pediatric Dentistry School of Dentistry of Ribeirão Preto University of São Paulo Ribeirão Preto São Paulo Brazil

**Keywords:** case reports, dentistry, oral health, phenotype, rare diseases

## Abstract

**Aims**: Developmental delay with gastrointestinal, cardiovascular, genitourinary, and skeletal abnormalities (DEGCAGS) syndrome (OMIM #619488) is a rare autosomal recessive disorder caused by pathogenic variants in ZNF699 and characterized by developmental delay and multisystem involvement. Although several systemic and craniofacial manifestations have been described, oral findings have not yet been reported. This case report describes the craniofacial and oral features of a patient with DEGCAGS syndrome, contributing to the expansion of its phenotypic spectrum.

**Methods and Results**: A 10‐year‐old girl with a homozygous pathogenic variant in ZNF699 was referred for dental evaluation. Clinical examination revealed hair thinning, dolichocephaly, retromicrognathia, synophrys, smooth philtrum, thin upper lip, increased overjet, and deep bite. Intraoral findings included generalized spacing, gingivitis, active carious lesions, hypomineralization of primary molars, talon cusps on the maxillary central incisors, erosive tooth wear, and signs consistent with sleep bruxism. Radiographic evaluation revealed taurodontism, particularly in teeth 16 and 26, shortened roots in the mandibular incisors, and increased pericoronal space associated with developing teeth.

**Conclusion**: These findings expand the oral phenotype of DEGCAGS syndrome and highlight the importance of dental evaluation in patients with rare genetic disorders. Further reports are needed to better characterize the oral manifestations associated with this condition.

## 1 Introduction

Developmental delay with gastrointestinal, cardiovascular, genitourinary, and skeletal abnormalities (DEGCAGS) syndrome (OMIM #619488) is a rare autosomal recessive genetic disorder characterized by developmental delay and multisystem involvement. The condition was first described in 2021, when Bertoli‐Avella et al. [1] reported 13 individuals from 12 unrelated consanguineous families carrying pathogenic variants in the ZNF699 gene, presenting a complex neurodevelopmental disorder with multisystem involvement. In this cohort, patients exhibited coarse facial features, often accompanied by microcephaly, as well as prematurely graying hair [1]. Reported dysmorphic features included low anterior hairline, thick hair, hirsutism, dense eyebrows, synophrys, long eyelashes, elongated palpebral fissures, proptosis, strabismus, a bulbous nose with a low columella, low‐set ears, smooth philtrum, wide mouth with protruding tongue, thin upper lip, micrognathia, and short neck [1]. All individuals presented severe global developmental delay with significant intellectual impairment and hypotonia; however, details regarding independent ambulation and speech development were limited in the clinical descriptions^1^.

In 2022, Biela et al. [2] reported the 14th case with a variant in the same gene, with clinical features largely consistent with the original findings. Based on these 14 cases and an additional patient identified in a hospital in the United States, Freeman et al. [3] developed a next‐generation phenotyping workflow to more comprehensively characterize the phenotypic spectrum of the syndrome. Later in 2023, three additional cases were published [4, 5], including one patient with ocular abnormalities extensively documented by high‐resolution retinal imaging.

In 2024, Karimi et al. [6] substantially expanded current knowledge by compiling clinical and genetic data from 30 individuals—12 of whom were newly reported—to explore the genotype–phenotype spectrum and investigate the presence of a potential DNA methylation episignature that could support the diagnosis of this highly variable condition lacking pathognomonic features. In 2025, additional reports further contributed to the literature, including hematological findings in a patient evaluated for suspected inherited bone marrow failure [7] and respiratory challenges related to the management of laryngeal hamartomas in an infant [8].

In view of these recent advances, the present study aims to describe the craniofacial and, for the first time, the orodental features of a patient with DEGCAGS syndrome, representing the first reported case in Brazil. The documentation of these findings expands the phenotypic spectrum associated with pathogenic variants in ZNF699 and provides additional insight into the oral manifestations of this rare genetic condition. This case report was prepared in accordance with the CARE (CAse REport) guidelines to ensure transparency and completeness in clinical reporting [9].

## 2 Case Report

**FIGURE 1:**
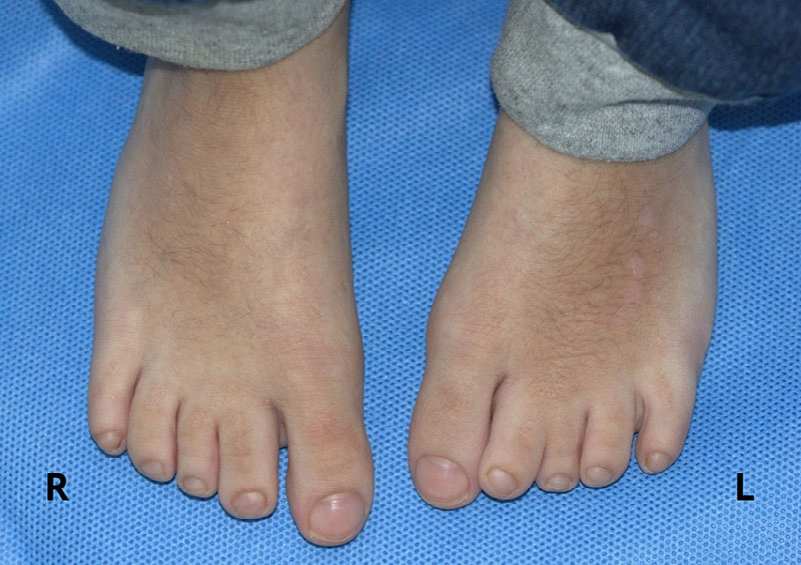
Clinical appearance of the patient's feet, showing syndactyly between the second and third toes of the left foot.

A 10‐year‐old girl accompanied by her family was referred to the Special Care Dentistry Clinic at the School of Dentistry of Ribeirão Preto, University of São Paulo, for specialized dental evaluation and management. She had been previously diagnosed with DEGCAGS syndrome based on molecular analysis, which identified a homozygous pathogenic variant in the ZNF699 gene located on chromosome 19p13. Her medical history revealed neuropsychomotor developmental delay, with complete dependence on her caregiver for activities of daily living. According to a medical report issued by the Hospital das Clínicas of the School of Medicine of the same institution, where she receives regular follow‐up care, the patient also presented with low weight and short stature, a history of intestinal malrotation corrected surgically, and dysmorphic features including cutis marmorata and syndactyly between the second and third toes of the left foot ([]Figure 1).

**FIGURE 2:**
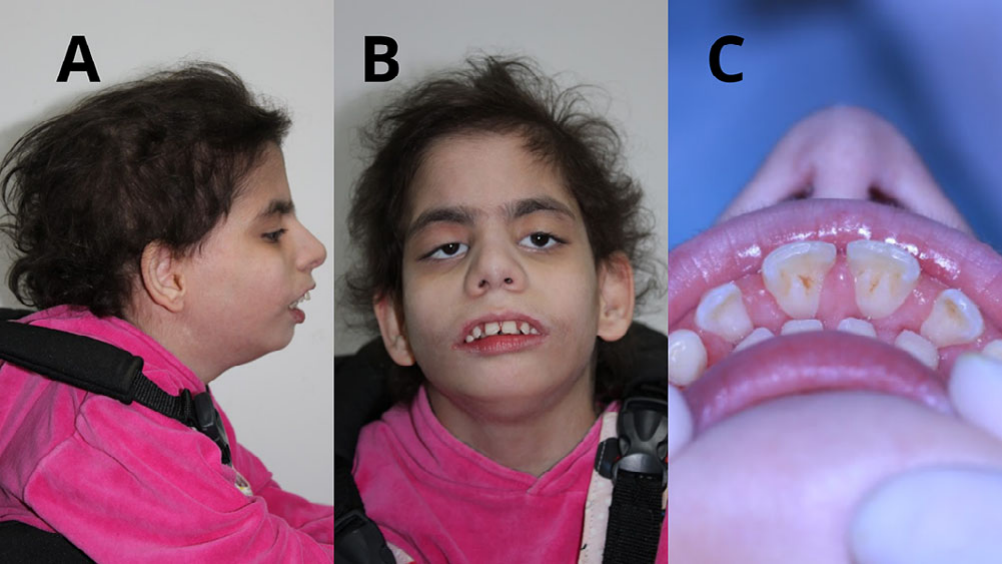
Craniofacial and occlusal characteristics of the patient. (A) Lateral view showing hair thinning, dolichocephalic facial pattern, and retromicrognathia. (B) Frontal view showing synophrys, thin upper lip, and smooth philtrum. (C) Intraoral view showing increased overjet and deep bite, with contact of the mandibular incisors with the palate.

During the extraoral clinical examination (Figure 2), craniofacial features consistent with those previously reported in the syndrome [1] were observed. These included hair thinning, dolichocephaly, mandibular retrognathism associated with micrognathia, and increased overjet. The patient also presented synophrys, characterized by the continuity of hair in the glabellar region, giving the appearance of a single eyebrow. The philtrum appeared smooth, lacking the usual vertical ridges, and the upper lip was thin. A deep bite was also observed, in which the mandibular incisors were in direct contact with the palate rather than with the palatal surfaces of the maxillary incisors.

During anamnesis, it was reported that the patient was under continuous use of Esio 20 mg (esomeprazole magnesium) due to gastroesophageal reflux disease (GERD), gastritis, and esophagitis; Fortini Plus as a hypercaloric pediatric supplement; Folacin (folic acid); a probiotic containing 20 billion CFU; milk of magnesia; and vitamin D3. Clinical examination revealed reduced salivary flow, dental erosion consistent with a history of reflux, and frequent tongue coating. Chronic constipation and episodes of regurgitation were also reported, corroborating the medical and medication history.

**FIGURE 3:**
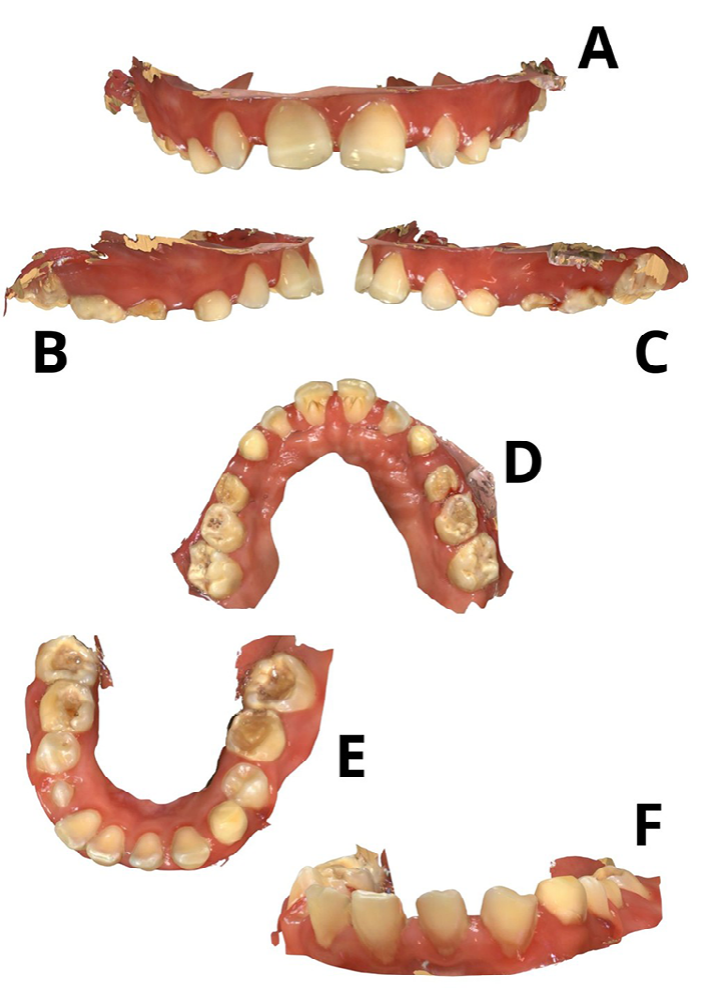
Intraoral scans used to document the patient's intraoral characteristics. (A) Frontal view of the maxillary arch. (B) Right lateral view of the maxillary arch. (C) Left lateral view of the maxillary arch. (D) Occlusal view of the maxillary arch. (E) Occlusal view of the mandibular arch. (F) Frontal view of the mandibular arch. Generalized diastemata are observed in both dental arches, as well as carious lesions in primary and permanent molars, talon cusps in the maxillary central incisors, findings consistent with enamel hypomineralization in the maxillary first primary molars, and incisal wear in the anterior teeth.

Although the patient cooperated adequately during the intraoral clinical examination, she demonstrated limited tolerance to photographic documentation, mainly due to discomfort caused by intraoral mirrors and the air spray from the dental syringe. To overcome this limitation and ensure greater comfort, dental records were obtained using intraoral scanning, a technique well accepted by the child that allowed detailed documentation of her oral condition (Figure 3). The examination revealed generalized diastemata in both dental arches, as well as gingivitis associated with poor oral hygiene. Active carious lesions were identified in the second primary molars and first permanent molars, the latter also showing a wear pattern consistent with erosive tooth wear. The maxillary first primary molars (teeth 54 and 64) exhibited changes consistent with enamel hypomineralization. The maxillary central incisors (teeth 11 and 21) presented talon cusps, a developmental anomaly characterized by an enamel projection in the cingulum region of the palatal surface. Additionally, incisal wear of the anterior teeth was observed, consistent with the caregiver's report of sleep bruxism.

**FIGURE 4:**
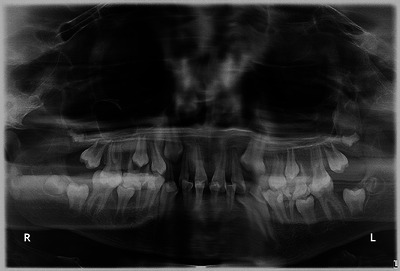
Panoramic radiograph showing mixed dentition. Taurodontism is observed, particularly in teeth 16 and 26, characterized by enlarged pulp chambers and apical displacement of the root furcation. Shortened roots are also evident in the mandibular permanent incisors (teeth 31, 32, 41, and 42). Increased pericoronal space is associated with teeth 13 and 45.

To complement the evaluation of the mixed dentition and investigate possible dentoalveolar alterations, a panoramic radiograph was obtained (Figure 4). The examination revealed taurodontic morphology, most evident in teeth 16 and 26, characterized by enlarged pulp chambers and apical displacement of the root furcation. Shortened roots were also observed in the mandibular permanent incisors (teeth 31, 32, 41, and 42), as well as increased pericoronal space associated with teeth 13 and 45.

Although some craniofacial features of this condition have been previously reported in the literature, the concomitant identification of oral alterations (as observed in this case) represents a relevant and previously unreported finding. This combination reinforces the novelty of this report and expands the phenotypic understanding of the condition.

A dental treatment plan was established based on the clinical and radiographic findings, focusing on preventive and restorative care. Oral hygiene instructions were provided to the caregiver, topical fluoride therapy was indicated, active carious lesions were scheduled for restorative treatment, and the hypomineralized teeth were placed under periodic monitoring. In addition, the patient was referred for orthodontic evaluation due to increased overjet, deep bite, and mandibular retrognathia. The patient is currently under follow‐up, and no long‐term outcomes are available at this stage.

Overall, the patient's clinical course includes preterm birth, neonatal intensive care unit admission, diagnosis of DEGCAGS syndrome through genetic testing, surgical correction of intestinal malrotation, and referral for specialized dental care.

## 3 Discussion

This case aimed to describe previously recognized craniofacial features and, for the first time, to document oral findings associated with a recently described rare genetic condition [1]. Given the scarcity of available evidence, the marked phenotypic variability, and the absence of pathognomonic features, the detailed description of this case contributes to expanding the clinical spectrum of the syndrome and supports diagnosis by both medical and dental teams.

Infant sleeping position significantly influences cranial shape [10]. Newborns requiring intensive care, particularly preterm infants, are at increased risk of developing deformities such as positional plagiocephaly and dolichocephaly, characterized by an anteroposterior elongation of the head [10]. In addition to positional etiology, dolichocephaly may result from craniosynostosis, a condition caused by premature fusion of one or more cranial sutures [11]. When syndromic, craniosynostosis is frequently associated with craniofacial dysmorphisms, skeletal abnormalities, neurological impairment, and developmental delay [11], features that overlap with those reported in DEGCAGS syndrome. In the present case, although dolichocephaly was observed, no diagnosis of craniosynostosis has been established. A positional etiology remains plausible considering the patient's preterm birth and prolonged neonatal intensive care unit stay, both recognized risk factors for acquired cranial deformation [10].

Breastfeeding provides well‐established nutritional, immunological, and developmental benefits [12]. It has also been described as a form of “early functional orthopedics” because it promotes coordinated activation of masticatory muscles and anterior traction forces on the mandible [13, 14, 15]. In this case, breastfeeding was discontinued after 15 days due to neonatal lactose intolerance, malnutrition, and difficulty maintaining weight. As an alternative, lactose‐free milk was administered via tube feeding. In this context, the absence of functional stimuli associated with breastfeeding may have contributed—although not in isolation—to the observed craniofacial growth pattern, particularly retromicrognathia, increased overjet, and generalized spacing.

Facial features such as synophrys, smooth philtrum, and thin upper lip are part of a set of dysmorphisms previously reported in individuals with pathogenic variants in ZNF699^1^, reinforcing the craniofacial phenotype associated with DEGCAGS syndrome. Synophrys has been associated with disturbances in neural crest cell migration and differentiation, an embryonic structure that also contributes to the formation of dental tissues and much of the craniofacial skeleton [16]. Likewise, a smooth philtrum and thin upper lip may reflect alterations in midfacial morphogenesis during embryogenesis [17]. Together, these findings support a recognizable facial pattern and contribute to refining the phenotypic spectrum of the syndrome.

Continuous medication use, combined with GERD, may have contributed to the observed oral alterations. Patients with reflux often exhibit reduced salivary flow, decreased buffering capacity, and lower salivary pH, all of which favor enamel demineralization [18]. In addition, carbohydrate‐rich hypercaloric supplements, particularly when associated with poor oral hygiene, increase the risk of dental caries. These factors likely contributed to the erosive tooth wear and carious lesions observed in this patient.

Hypomineralization of primary molars is a multifactorial condition associated with systemic factors and genetic predisposition during enamel formation [19]. Hypomineralized molars with yellow or brown opacities show approximately 20%–22% lower mineral density compared with sound teeth [20]. These defects are frequently associated with post‐eruptive enamel breakdown [21], which may increase the risk of dentin caries by up to tenfold [22]. In the present case, post‐eruptive breakdown facilitated biofilm accumulation and the development of atypical carious lesions, reinforcing the importance of early diagnosis and continuous dental monitoring.

Talon cusps observed on the maxillary central incisors represent a developmental dental anomaly resulting from disturbances during the morphodifferentiation stage. They are characterized by an enamel projection in the cingulum region and may contain dentin and, occasionally, pulp tissue [23, 24]. This anomaly has been associated with genetic syndromes and neurodevelopmental disorders, suggesting the involvement of common embryological mechanisms, including epithelial–mesenchymal interactions and neural crest cell dynamics^2^ ^3^.

Sleep bruxism is characterized by repetitive masticatory muscle activity during sleep and has a multifactorial etiology involving biological, psychosocial, environmental, and genetic factors [25, 26, 27]. Conditions associated with an increased risk include respiratory disorders [28] and GERD [29]. In the present case, the patient's reflux history may have contributed to the reported nocturnal bruxism, which is clinically relevant because of its potential impact on dental structures.

Taurodontism, most evident in teeth 16 and 26, is a morphological anomaly characterized by enlargement of the pulp chamber and apical displacement of the furcation due to alterations in Hertwig's epithelial root sheath during root development [24, 30]. Although it may occur in isolation, it has been associated with several genetic syndromes [30]. Shortened roots were also observed in the mandibular incisors, suggesting additional developmental alterations affecting root formation. Increased pericoronal space associated with teeth 13 and 45 may reflect alterations in dental development or eruption dynamics. Although frequently representing a variation of the dental follicle, enlarged pericoronal spaces may also correspond to pathological entities requiring appropriate diagnosis and follow‐up [31]. Together, these radiographic findings broaden the characterization of oral manifestations associated with DEGCAGS syndrome.

Although the biological role of ZNF699 remains incompletely understood, the coexistence of multiple developmental dental anomalies in the present patient—including enamel hypomineralization, talon cusps, taurodontism, shortened roots, increased overjet, and generalized spacing—raises the possibility that pathogenic variants in this gene may affect distinct stages of craniofacial and dental development. Given the rarity of DEGCAGS syndrome and the absence of previous reports focusing on oral manifestations, no causal relationship can currently be established. Nevertheless, these findings provide preliminary evidence that warrants further investigation in future cases.

As a case report, this study presents inherent limitations, including the inability to establish causal relationships and the absence of longitudinal follow‐up. Nevertheless, it contributes to expanding the phenotypic spectrum associated with variants in the ZNF699 gene, particularly regarding oral manifestations that have not been previously described, and highlights the importance of dental evaluation in the management of patients with rare genetic syndromes.

The patient's caregiver reported satisfaction with the dental evaluation and expressed understanding of the importance of oral health care in the context of the patient's systemic condition.

## 4 Conclusion

This single case report suggests that craniofacial and oral manifestations may be part of the clinical spectrum associated with pathogenic variants in the ZNF699 gene. The documentation of previously recognized craniofacial features together with novel oral findings contributes to expanding the phenotypic characterization of DEGCAGS syndrome and may assist healthcare professionals in the recognition of similar cases. Further reports are needed to confirm and better define the oral phenotype associated with this condition. Increased awareness of the importance of dental evaluation in genetically diagnosed patients may contribute to more comprehensive multidisciplinary care for individuals with rare genetic disorders.

## Author Contributions

Conception and planning of the case: JP, LBF, JTAJ; Collection, analysis, and interpretation of data: JP, LBF, JTAJ, RABS, KVDS, FWGPS, FKC; Elaboration or revision of the manuscript: JP, KFM, AMQ, FKC; Approval of the final version: FWGPS, AMQ, FKC; Public responsibility for article content: FKC.

## Consent

Written informed consent was obtained from the patient's legal guardian for publication of this case report.

## Conflicts of Interest

The authors declare no conflicts of interest.

## Data Availability

Data sharing not applicable – no new data generated, or the article describes entirely theoretical research.
